# Dr. Walter W. Johnson

**Published:** 1912-03

**Authors:** 


DR. WALTER W. JOHNSON of Rochester, died Jan. 26, aged 83. He was graduated from the N. Y. Homeopathic College in 1887 and was a charter member of the Roentgen Ray Society of America.

